# Quantitative resistance to clubroot infection mediated by transgenerational epigenetic variation in Arabidopsis

**DOI:** 10.1111/nph.15579

**Published:** 2018-11-30

**Authors:** Benjamin Liégard, Victoire Baillet, Mathilde Etcheverry, Evens Joseph, Christine Lariagon, Jocelyne Lemoine, Aurélie Evrard, Vincent Colot, Antoine Gravot, Maria J. Manzanares‐Dauleux, Mélanie Jubault

**Affiliations:** ^1^ IGEPP INRA AGROCAMPUS OUEST Université de Rennes F‐35000 Rennes France; ^2^ Institut de Biologie de l'Ecole Normale Supérieure (IBENS) Ecole Normale Supérieure Centre National de la Recherche Scientifique (CNRS) Institut National de la Santé et de la Recherche Médicale (INSERM) F‐75005 Paris France

**Keywords:** *Arabidopsis*, clubroot, epigenetics, EpiRIL, methylome, *Plasmodiophora brassicae*, quantitative resistance, transgenerational

## Abstract

- Quantitative disease resistance, often influenced by environmental factors, is thought to be the result of DNA sequence variants segregating at multiple loci. However, heritable differences in DNA methylation, so‐called transgenerational epigenetic variants, also could contribute to quantitative traits. Here, we tested this possibility using the well‐characterized quantitative resistance of Arabidopsis to clubroot, a *Brassica* major disease caused by *Plasmodiophora brassicae*.
- For that, we used the epigenetic recombinant inbred lines (epiRIL) derived from the cross *ddm1‐2* × Col‐0, which show extensive epigenetic variation but limited DNA sequence variation. Quantitative loci under epigenetic control (QTL ^epi^) mapping was carried out on 123 epiRIL infected with *P. brassicae* and using various disease‐related traits.
- EpiRIL displayed a wide range of continuous phenotypic responses. Twenty QTL ^epi^ were detected across the five chromosomes, with a *bona fide* epigenetic origin for 16 of them. The effect of five QTL ^epi^ was dependent on temperature conditions. Six QTL ^epi^ co‐localized with previously identified clubroot resistance genes and QTL in Arabidopsis.
- Co‐localization of clubroot resistance QTL ^epi^ with previously detected DNA‐based QTL reveals a complex model in which a combination of allelic and epiallelic variations interacts with the environment to lead to variation in clubroot quantitative resistance.

## Introduction

Clubroot caused by the protist *Plasmodiophora brassicae* is a major disease of *Brassicaceae* including the three most economically important *Brassica* species, *B. napus*,* B. rapa* and *B. oleracea* (Dixon, 2009), and the model plant *Arabidopsis thaliana* (Koch *et al*., 1991). Infection with *P. brassicae* leads to tumorous club formation on roots resulting from cell hyperplasia and hypertrophy (Ingram & Tommerup, 1972). Cropping practices and crop protection products have limited success in controlling clubroot (Dixon, 2009). Currently, one of the most effective ways to limit the impact of this disease is to use resistant varieties (Diederichsen *et al*., 2009). To date, both qualitative and quantitative trait loci (QTL) for clubroot resistance have been identified in different *Brassicaceae* species (Manzanares‐Dauleux *et al*., 2000a; Rocherieux *et al*., 2004; Alix *et al*., 2007; Jubault *et al*., 2008; Piao *et al*., 2009; Lee *et al*., 2016). Current approaches to generating resistant varieties rely mainly on a few loci controlling qualitative resistance, with the inevitable outcome of rapid adaptation of the pathogen populations (Diederichsen *et al*., 2009). In this context, diversification and access to other sources of clubroot resistance variability is becoming necessary.

Numerous studies (Dowen *et al*., 2012; Luna *et al*., 2012; Zhang *et al*., 2013; Liu *et al*., 2015; Aoun *et al*., 2017; Zheng *et al*., 2017) have reported that plant responses to abiotic (temperature, drought) and biotic stresses could be associated with epigenetic variation in addition to nucleotidic variation. For instance, Dowen *et al*. (2012) and Yu *et al*. (2013) have shown that Arabidopsis mutants altered in the maintenance of DNA methylation in the CG, CHG and CHH contexts, showed strong resistance to *Pseudomonas syringae* pv *tomato* strain DC3000. The role of epigenetics in the expression of adaptive plant traits thus suggests that epigenetic variability could be used for generating stress‐tolerant or resistant plants. Furthermore, the occurrence of natural DNA methylation variants (epialleles) in plants (Becker *et al*., 2011; Schmitz *et al*., 2011) and their implication in evolution (Weigel & Colot, 2012) suggest that epialleles could be considered as a source of variability in plant breeding. In this ‘epigenetic breeding’ approach (Gallusci *et al*., 2017), two conditions are needed: transgenerational inheritance of epialleles and a clear connection between epigenotype and observed phenotype. Previous studies demonstrated that epialleles could be stably transmitted across at least eight generations (Johannes *et al*., 2009; Teixeira *et al*., 2009), and that such heritable differences in DNA methylation could be associated with heritable phenotypic variation for several complex traits (Johannes *et al*., 2009; Reinders *et al*., 2009). However, linking heritable phenotypic variation to epigenetic variation remains challenging because of the difficulty in teasing apart its effects to that of DNA sequence variation in natural settings (Johannes *et al*., 2008; Quadrana & Colot, 2016). This problem can, however, be greatly alleviated in Arabidopsis by using experimental populations of so‐called epigenetic recombinant inbred lines (epiRIL), which show extensive epigenetic variation but limited DNA sequence variation (Johannes *et al*., 2009; Reinders *et al*., 2009). One such population (Johannes *et al*., 2009) was indeed used to build a genetic map based solely on heritable differences in DNA methylation (differentially methylated regions, DMR) (Colomé‐Tatché *et al*., 2012) and to identify epigenetic QTL (QTL^epi^) for several complex traits (Cortijo *et al*., 2014; Kooke *et al*., 2015; Aller *et al*., 2018).

In the present study, we used this same epiRIL population to determine the impact of heritable differences in DNA methylation on the response of Arabidopsis to clubroot. We first showed that *ddm1* mutants were less susceptible to clubroot than the wild‐type Col‐0 and that the assessed subset of 123 epiRIL displayed a wide range of continuous phenotypic responses to clubroot. Twenty QTL^epi^ were detected across the five chromosomes, with a *bona fide* epigenetic origin for 16 of them. We have thus demonstrated that heritable differences in DNA methylation also could contribute to quantitative resistance to clubroot. Six QTL^epi^ co‐localized with previously identified clubroot resistance genes and QTL in Arabidopsis, revealing that quantitative resistance to clubroot in natural accessions could be controlled by both nucleotidic and epigenetic variations.

## Materials and Methods

### Plant material

#### Plant stocks

Mutant plants altered in genes encoding chromatin modifiers were ordered from the NASC. All plants were obtained from T‐DNA insertion in the Columbia (Col‐0) genetic background and showed a homozygous insertion in the plant genome. The mutant lines used were *drm2‐2* (SALK_150863), *hda 15* (SALK_004027C), *atxr5* (SALK_130607C), *hac1* (SALK_082118C), *srt2* (SALK_149295C) and *ddm1* (SALK_000590C). T‐DNA insertions and homozygosity were confirmed by PCR using the set of appropriate primers designed with the T‐DNA Primer Design interface (http://signal.salk.edu/tdnaprimers.2.html, Supporting Information Table S1). The epigenetic recombinant inbred lines (epiRIL) population is that derived from a cross between the *Arabidopsis thaliana* ecotype Col‐0 and a Col‐0 line carrying the *ddm1‐2* mutant allele (Johannes *et al*., 2009). Note that the *ddm1‐2* allele was obtained by EMS mutagenesis, not T‐DNA transformation (Vongs *et al*., 1993). EpiRIL population seeds were obtained from the Versailles Arabidopsis Stock Center (http://publiclines.versailles.inra.fr/) at the Institute Jean‐Pierre Bourgin.

### Clubroot pathogen

All clubroot tests were performed with the *Plasmodiophora brassicae* eH isolate described by Fahling *et al*. (2003). Isolate eH belongs to the P1 pathotype according to the classification using the differential host set of Some *et al*. (1996). One millilitre of resting spore suspension (10^7^ spores ml^−1^) prepared according to Manzanares‐Dauleux *et al*. (2000b) was used for pathogen inoculation 10 d after germination (stage 1.04; Boyes *et al*., 2001). The inoculum was applied to the bottom of the stem base of each seedling.

### Growth conditions

The Arabidopsis accession Col‐0 and the six mutant lines (Col‐0 background) were evaluated in a randomized complete block design (with three blocks) against the eH *P. brassicae* isolate. For QTL^epi^ detection, the 123 epiRIL that have been epigenotyped previously (Colomé‐Tatché *et al*., 2012), together with the two parental lines, were phenotyped in four biological replicates, split in two growth rooms, in a randomized complete block design (with two blocks per replicate and six plants per epigenotype per block). In growth room‐2, four temperature sensors per block were placed at the height of plants. For each pathological test, seed germination was synchronized by placing seeds on wet blotting paper in Petri dishes for 2 d at 4°C. Seeds were sown individually in pots (4 cm diameter) containing a sterilized mix (by autoclaving) composed of 2/3 compost and 1/3 vermiculite. Per block, six plants per genotype (T‐DNA mutants, Col‐0, *ddm1‐2* and epiRIL) were grown in controlled conditions of 16 h light (110 μmol m^−2 ^s^−1^) at 21°C and 8 h dark at 18°C.

### Phenotyping

Phenotyping was performed 3 wk after inoculation (21 d post‐inoculation (dpi)). Plants were thoroughly rinsed with water and photographed. Infected roots were removed and frozen in liquid nitrogen. For the epiRIL and their parents, four disease‐related traits were assessed: longest leaf length (Lfi), disease index (DI), root biomass (Rbi) and pathogen DNA quantity (Pb), whereas for mutants, only DI was evaluated. DI was calculated according to Manzanares‐Dauleux *et al*. (2000b) and pathogen DNA quantification was determined by quantitative polymerase chain reaction (qPCR). These phenotypic traits were chosen due to their relevance for the study of the quantitative response to eH isolate on Arabidopsis (Jubault *et al*., 2008; Gravot *et al*., 2011; Lemarié *et al*., 2015a,b). The longest leaf length (Lfni) and the root biomass (Rbni) were also measured at 28 d post sowing on noninfected plants cultivated in growth room‐2 in a randomized complete block design with 24 plants per (epi)genotype. Two new variables were then obtained by calculating the difference between these ‘control’ values with those obtained on infected plants; these new variables were termed ΔLf and ΔRb.

### Pathogen DNA quantification

Total genomic DNA (plant and pathogen) was extracted from lyophilized infected roots with the NucleoSpin Plant II kit according to manufacturer's instructions. After the evaluation of DNA concentration by Nanodrop ND‐1000, 5–10 ng of total genomic DNA extract and specific primers were used to quantify by qPCR pathogen the DNA quantity in each epiRIL and the parental lines. The pathogen‐specific primers designed on the 18S rRNA of *P. brassicae* used were PbF‐K1 5′ TTGGGTAATTTGCGCGCCTG 3′; PbR‐K1 5′ CAGCGGCAGGTCATTCAACA 3′. qPCR reactions were carried out in a thermocycler (LightCycler 480 II Roche), with Syber green (LightCycler^®^ 480 SYBR Green I Master). The qPCR conditions used were: 50 cycles of denaturation at 95°C for 15 s, annealing/extension at 63°C for 30 s and 72°C for 30 s. Absolute quantification of pathogen DNA was carried out using a standard curve obtained based on a series of known amounts of pathogen DNA. The average of the pathogen quantity present in each epiRIL (ratio of pathogen DNA quantity to total DNA used for qPCR) was used in further statistical analyses and QTL^epi^ detection.

### Data analysis

Two generalized linear models (glm) were used to determine the effects of epigenome, temperature and epigenome × temperature interaction with R/glm2 (Marschner, 2011) in R v.3.2.2 (R Development Core Team, 2015). For each model, the family distribution option of the glm function was adapted according to the data distribution. The first glm (glm1) described in Eqn 1 was used to estimate the epigenotype effect of each epiRIL across biological replicates in each growth room:

(Eqn 1) $$Y_{\mathrm{ijk}}=\mu +G_{i}+R_{j}+B_{k}\left(R_{j}\right)+e_{\mathrm{ijk}}$$

where *μ*, mean general effect; *G*_*i*_, differential effect between epigenotypes; *R*_*j*_, differential effect between replicates; *B*_*k*_ (*R*_*j*_), interaction between blocks and replicates; and *e*_*ij*_, residual variance. Based on this model, broad sense heritability was estimated using the following equation:

(Eqn 1.1) $$H^{2}=\frac{\sigma_{G}^{2}}{\sigma_{G}^{2}+\left(\frac{\sigma_{e}^{2}}{n}\right)}$$

($\sigma_{G}^{2}$, estimated epigenetic variance; $\sigma_{e}^{2}$, estimated environmental variance; and *n*, number of replicates per line).

The second generalized linear model (glm2) described in Eqn (Eqn 2) was used to estimate the epigenotype × temperature effect in growth room‐2 for each epiRIL across the biological replicates:

(Eqn 2) $$Y_{\mathrm{ijkl}}=\;\mu +G_{i}+T_{j}+R_{k}+B_{l}\left(R_{k}\right)+GT_{\mathrm{ij}}+TR_{\mathrm{jk}}+TB{\left(R\right)}_{\mathrm{jlk}}+e_{\mathrm{ijkl}}$$

where *μ*, mean general effect; *G*_*i*_, differential effect between epigenotypes; *T*_*j*_, differential temperature conditions; *R*_*k*_, differential effect between replicates; *B*_*l*_ (*R*_*k*_), interaction between blocks and replicates; *GT*_*ij*_, interaction between epigenotype and temperature condition; *TR*_*jk*_, interaction between replicate and temperature condition; *TB*(*R*)_*jlk*,_ interaction between block and temperature condition; and *e*_*ijkl*_, residual variance). Based on this model, broad sense heritability was estimated using the following equation:

(Eqn 2.1) $$H^{2}=\frac{\sigma_{G}^{2}}{\sigma_{G}^{2}+\sigma_{\mathrm{GT}}^{2}+\left(\frac{\sigma_{e}^{2}}{n}\right)}$$

where $\sigma_{G}^{2}$, estimated epigenetic variance; $\sigma_{\mathrm{GT}}^{2}$, estimated epigenetic × temperature variance; $\sigma_{e}^{2}$, estimated environmental variance; and *n*, number of replicates per line.

For all traits, least square means on each effect according to the two models described above were estimated with the function lsmeans of the R/lsmeans package (,Lenth 2016) in R v.3.2.2 (R Core Team, 2015). The lsmeans function also was used to extract the epigenotype effect (*G*) and the interaction epigenotype × temperature (*GT*) of each trait according to the generalized model used. Differences in longest length leaf (Δ*Lf*) and root biomass (Δ*Rb*) between infected and control plants were calculated from *G*.

### QTL^epi^ detection

The G and GT of each trait were treated with the package R/qtl (Broman *et al*., 2003) in R v.3.2.2 (R Development Core Team, 2015). The package snow in R (Tierney *et al*., 2015) allowing the use of processor cores as cluster was used to reduce permutation calculation time. Simple Interval Mapping (SIM) was first carried out to identify potential QTL^epi^ with the Haley–Knott (hk) method (Broman & Sen, 2009), using a step size of 2 cM and a window size of 10 cM. One thousand permutations with the hk method were carried out in order to determine SIM threshold levels for each condition and trait analysed. The significance level of threshold was fixed at *α* = 0.05. In order to integrate the possibility of the presence of multiple QTL^epi^ on the same chromosome, a manual multiple QTL mapping (MQM) approach (Broman & Sen, 2009) was used based on the results of SIM analysis. For this, the stepwise function was used in order to select the QTL^epi^ (forward and backward system, option ‘additive.only = FALSE’) based on the preliminary putative QTL^epi^ identified by SIM. For each trait, a minimum of two potential QTL^epi^ was used in the stepwise function even if only one potential QTL^epi^ was detected in SIM. Logarithm of odds (LOD) thresholds were calculated using 1000 permutations with the function scantwo with a significance level of *α* = 0.05. Once the QTL^epi^ selected, the model was fitted (fitqtl function), in order to calculate the LOD scores and the percentage of variation explained by each and all QTL^epi^ (*R* ^2^). The confidence interval of each QTL^epi^ was calculated with the lodint function with a LOD drop of one parameter. The epiallele effect was evaluated with the function effectplot. Putative interactions among the QTL^epi^ incorporated in the model were tested with the function addint according to Broman & Sen (2009).

### DNA sequence variation

Whole genome sequence data are available for the 123 epigenotyped epiRIL (Gilly *et al*., 2014). The joint identification of small‐scale variants (single‐base substitutions and indels < 100 bp) was performed using GATK HaplotypeCaller on the whole‐genome sequencing data available for 122 epiRIL. Raw variant calls were then filtered following GATK Best Practice suggestions and additional scripts. All variants were visually inspected using IGV and a subset of the detected single nucleotide polymorphism (SNP) was validated by PCR. For the analysis of transposition events (TE), TE‐Tracker (Gilly *et al*., 2014) was used to identify nonreference TE insertions in 102 epiRIL of sufficient genomic coverage. Raw calls were then filtered using in‐house scripts. All of the detected new TE insertions were visually inspected using IGV and a fraction of them were validated by PCR. Shared small‐scale and TE insertion variants are defined as present in at least 25% of the population. In order to evaluate the impact of shared sequence variants on the heritable variation observed in the epiRIL in response to clubroot, we compared various QTL models as described by Kooke *et al*. (2015). Three different models were tested: (1) using all QTL peak epigenetic markers (MM); (2) using each shared DNA sequence variant (SNP, indel, TE insertion) located in the confidence interval instead of the epigenetic marker at the peak; and (3) using both peak epigenetic markers and the shared DNA sequence variants included in the confidence interval. QTL detected using the three models were compared. If the DNA‐based markers had a more significant effect than the peak QTL epigenetic markers, then the model fitted was considered to be better or identical to the model with the peak epigenetic markers.

## Results

### *ddm1* mutants have reduced susceptibility to *P. brassicae*

**Figure 1:**
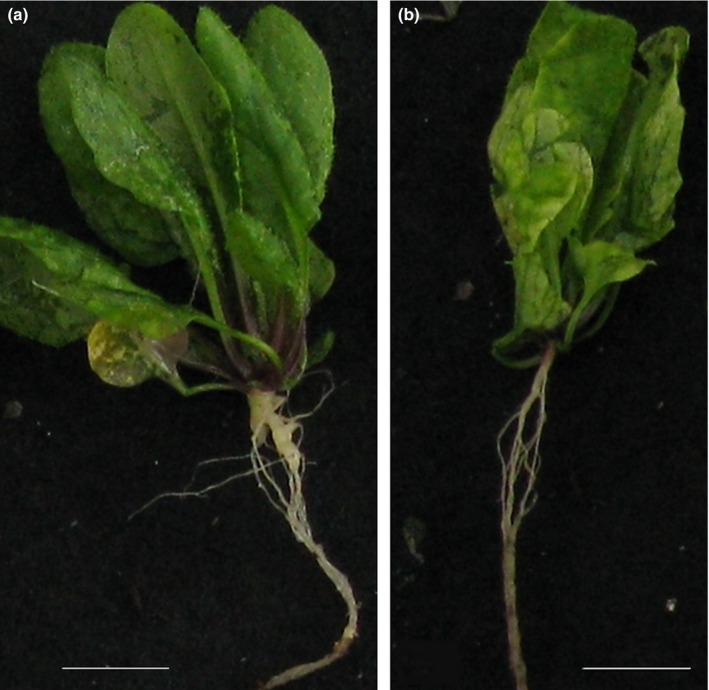
Clubroot symptoms shown by Col‐0 wild‐type and the *ddm1* mutant (Col‐0 background). Arabidopsis Col‐0 and mutants were inoculated 10 d after germination and phenotyped 3 wk post‐inoculation. (a) Col‐0 ecotype; (b) *ddm1* mutant. The *ddm1* mutant showed a significant decrease in disease symptoms (Dunett's test, *P* < 0.02) compared to Col‐0. Plant individuals are representative of standard observations made in our experimental conditions. Bars, 1 cm.

In order to determine if epigenetic variations could be associated with variations in response to clubroot infection, we assessed the response to infection of several Arabidopsis mutants affected in genes encoding chromatin modifiers. Infection of Col‐0 and six T‐DNA insertion mutants in genes encoding chromatin modifiers (*atxr5, ddm1, drm2, hac1, hdc15* and *srt2*) with the *P. brassicae* eH isolate led to a range of phenotypic responses. A statistically significant DI effect (ANOVA: *F* = 4.99, *P‐*value = 0.005) (Table S2a) was identified 21 dpi suggesting that epigenetics was involved in plant response to infection by *P. brassicae*. A Dunnett's post hoc test (Table S2) revealed a significant difference between *ddm1* and Col‐0 (*t* = −4.437, *P‐*value = 0.003) for the DI trait, *ddm1* showing reduced symptoms compared to Col‐0 (Fig. 1).

### Heritable differences in DNA methylation are associated with differential susceptibility to *P. brassicae*

#### EpiRIL response to *P. brassicae* is quantitative

**Figure 2:**
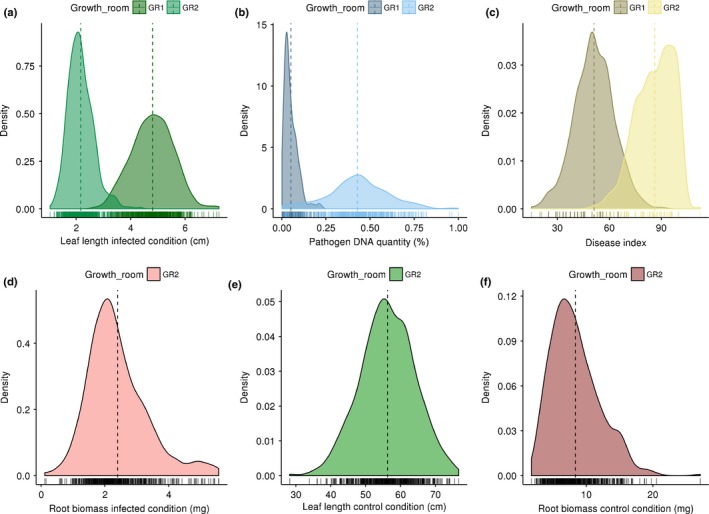
Distribution of phenotypic data measured in the two growth rooms used for pathological tests. For each trait studied, data are coloured to show in which growth chamber the Arabidopsis plants were grown. Vertical coloured dashed lines indicate means of traits in each growth room. Data distribution is shown for leaf length (a), pathogen DNA quantity (b), disease index (c) and root biomass (d) in infected condition, and for leaf length (e) and root biomass (f) in noninfected condition. GR1 corresponds to growth room‐1. GR2 corresponds to growth room‐2.

In order to identify epialleles involved in clubroot response, QTL^epi^ detection was carried out on the subset of 123 lines of the epiRIL population used previously in Colomé‐Tatché *et al*. (2012). In total, each genotype was assessed against *P. brassicae* isolate eH in four biological replicates, split in two growth rooms, each biological replicate being composed of two blocks. Distribution of the four disease‐related traits assessed on the 123 epiRIL showed continuous distribution suggesting polygenic control of these traits (Fig. 2). However, significant differences were observed for all traits between the biological replicates (Mann–Whitney *U*‐test) set up in the two growth rooms (DI: *W* = 3685.5, *P‐*value < 2.2e‐16; Lfi: *W* = 233 530, *P‐*value* *< 2.2e‐16*;* Pb: W = 4826, *P‐*value < 2.2e‐16), suggesting an influence of the growth conditions on the epiRIL response to clubroot (Table S3). Indeed, higher levels of disease symptoms were observed on the Col‐0 parent line (growth room‐1: DI = 53.25 ± 2.36; growth room‐2: DI = 90.75 ± 9.64) and on the epiRIL population (growth room‐1: DI = 51.17 ± 11.42; growth room‐2: DI = 86.27 ± 10.74) growing in growth room‐2 compared to growth room‐1. Consequently, we decided to analyse data from the two growth rooms independently. Analysis of biological replicates grown in each growth room showed a significant epigenotype effect (glm1, Eqn 1) for nearly all phenotypic traits measured (*P‐*value ranged from 0.02 to < 2.2e‐16 in growth room‐1, and from 0.35 to < 2.2e‐16 in growth room‐2; Table S4). Broad‐sense heritability (*H* ^2^) was estimated for each trait using Eqn 1.1 and ranged from 0.46 to 0.76 in the growth room‐1 and from 0.44 to 0.65 in the growth room‐2 depending on the trait studied (Table**** 1).

**Table 1:** Heritability estimates for each trait in the Arabidopsis epigenetic recombinant inbred lines population

Trait	Heritability model	*H* ^2^ Growth room‐1	*H* ^2^ Growth room‐2
DI	glm1	0.55	0.50
Pb	glm1	0.46	0.44
Lfi	glm1	0.76	0.65
Rbi	glm1	NA	0.62
DI	glm2	NA	0.50
Pb	glm2	NA	0.50
Lfi	glm2	NA	0.67
Rbi	glm2	NA	0.50

*H* ^2^ is the broad sense heritability calculated with the formula (Eqn 1): $H^{2}=\sigma_{G}^{2}/\left[\sigma_{G}^{2}+\left(\sigma_{e}^{2}/n\right)\right]$ and 2: $H^{2}=\sigma_{G}^{2}/\left[\sigma_{G}^{2}+\sigma_{\mathrm{GT}}^{2}+\left(\sigma_{e}^{2}/n\right)\right]$ including the variance of the temperature × epigenotype interaction. $\sigma_{G}^{2}$, the estimated epigenetic variance; $\sigma_{\mathrm{GT}}^{2}$, the estimated temperature × epigenetic interaction variance; $\sigma_{e}^{2}$, the estimated environmental variance; *n*, the number of replicates per line. DI, disease index; Pb, pathogen DNA quantity; Lfi, leaf length in infected condition; Rbi, root biomass in infected condition. NA, not available.

#### QTL analysis of data obtained in growth room‐1

**Figure 3:**
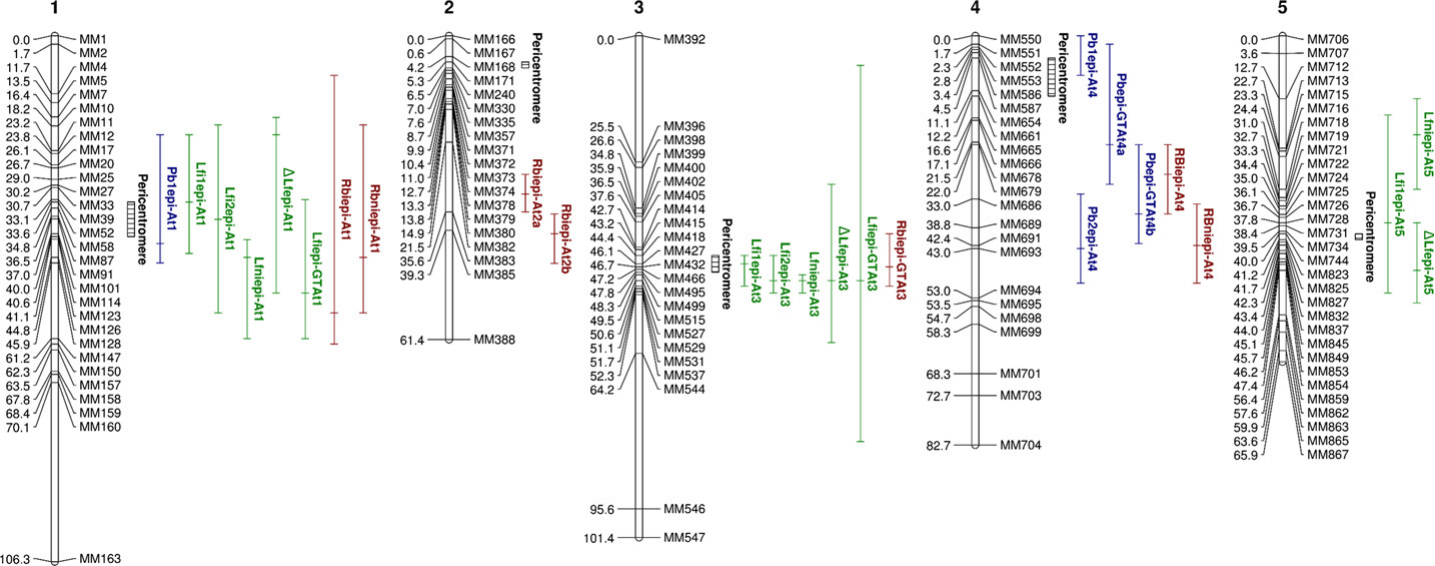
Epigenetic quantitative trait loci (QTL) identified in the epigenetic recombinant inbred lines (epiRIL) population in response to *Plasmodiophora brassicae* infection. Numbers above chromosomes indicate *Arabidopsis* chromosomes. MM indicates markers. For each marker, position in cM is indicated. The vertical bar length is equal to the one‐ logarithm of odds (LOD) likelihood confidence interval. The dash in the bar indicates the peak position. QTL name indicates the trait associated and chromosomal localization.

Phenotypic data measured on the two biological replicates set up in growth room‐1 were used in glm1 (Eqn 1) to extract the epigenotype effect (G) with the lsmean function. The epigenotype effect G identified for each trait was then used for the QTL^epi^ analysis. In total, five QTL^epi^ were detected (Fig.**** 3). Two QTL^epi^ were identified for Pb on chromosomes 1 and 4 (Pb1^epi^‐At1, Pb1^epi^‐At4) explaining 14.64% and 9.65% of the phenotypic variability, respectively. Three QTL^epi^ were detected for Lfi on chromosomes 1, 3 and 5 (Lfi1^epi^‐At1, Lfi1^epi^‐At3, Lfi1^epi^‐At5) explaining 15.49%, 15.01% and 8.11% of phenotypic variation, respectively (Table**** 2). The variance explained by each fitted QTL model was of 43.82% and 19.59% for Pb and Lfi, respectively. Surprisingly, no QTL^epi^ was identified for DI despite a significant effect of epigenotype on this trait. Confidence intervals of the QTL^epi^ detected ranged from 6.23 to 36 cM (Table 2). No epistatic interaction was found between QTL^epi^ for either trait. For the three QTL^epi^ detected for Lfi (markers nearest of the peak LOD score: MM52, MM427 and MM728), wild‐type (WT) epialleles were associated with an increase in the trait values. The *ddm1*‐derived epiallele was associated with an increase in pathogen quantity at QTL^epi^ on chromosome 1 (peak marker: MM123) whereas it was associated with a decrease in the Pb value on chromosome 4 (peak marker MM550) (Fig. S1; Table 2).

**Table 2:** Summary of epigenetic quantitative trait loci (QTL^epi^) detected in the Arabidopsis epigenetic recombinant inbred lines (epiRIL) population by multiple QTL mapping (MQM)

Trait	Condition	Test location	QTL	Chr	Pos	Lod	Closest peak marker	Confidence interval (cM)		*R* ^2^ (%)	*R* ^2^ for all QTL by trait	Favourable allele
G‐Rbi	Infected	Growth room‐2	Rbi^epi^‐At1	1	56.00	3.04	MM147	8.00	62.33	6.67	44.59	WT
		Growth room‐2	Rbi^epi^‐At2a	2	32.00	7.48	MM383	28.00	35.62	17.91		WT
		Growth room‐2	Rbi^epi^‐At2b	2	40.00	6.18	MM385	36.00	46.00	14.42		*ddm1‐2*
		Growth room‐2	Rbi^epi^‐At4	4	28.00	4.79	MM686	22.02	36.00	10.88		WT
G‐Lfi	Infected	Growth room‐1	Lfi1^epi^‐At1	1	33.60	6.51	MM 52	20.00	44.00	15.50	43.82	WT
		Growth room‐1	Lfi1^epi^‐At3	3	46.10	6.32	MM427	44.36	50.59	15.01		WT
		Growth room‐1	Lfi1^epi^‐At5	5	37.80	3.60	MM728	16.00	52.00	8.11		WT
		Growth room‐2	Lfi2^epi^‐At1	1	37.05	5.14	MM91	18.00	56.00	15.65	26.16	WT
		Growth room‐2	Lfi2^epi^‐At3	3	49.45	4.04	MM515	44.37	52.00	12.06		WT
G‐Pb	Infected	Growth room‐1	Pb1^epi^‐At1	1	42.00	4.36	MM123	20.00	45.93	14.64	19.59	WT
		Growth room‐1	Pb1^epi^‐At4	4	0.00	2.95	MM550	0.00	8.00	9.65		*ddm1‐2*
		Growth room‐2	Pb2^epi^‐At4	4	42.99	3.05	MM693	32.00	50.00	10.86	10.86	*ddm1‐2*
GT‐Lfi	Infected	Growth room‐2	Lfi^epi^GT‐At1	1	52.00	3.32	MM128	33.07	61.20	10.67	19.34	WT
		Growth room‐2	Lfi^epi^GT‐At3	3	49.45	2.47	MM547	6.00	82.00	7.81		WT
GT‐Rbi	Infected	Growth room‐2	Rbi^epi^GT‐At3	3	46.65	2.52	MM432	40.00	50.59	9.06	9.06	WT
GT‐Pb	Infected	Growth room‐2	Pb^epi^GT‐At4a	4	22.00	2.38	MM679	1.73	30.00	8.04	15.89	*ddm1‐2*
		Growth room‐2	Pb^epi^GT‐At4b	4	36.00	4.50	MM689	22.02	42.00	15.88		*ddm1‐2*
G‐Lfni	Noninfected	Growth room‐2	Lfni^epi^‐At1	1	44.80	8.32	MM126	41.15	61.20	19.66	46.20	WT
		Growth room‐2	Lfni^epi^‐At3	3	49.46	5.75	MM515	48.32	52.00	12.93		WT
		Growth room‐2	Lfni^epi^‐At5	5	20.00	4.76	MM713	12.73	31.00	10.49		WT
G‐Rbni	Noninfected	Growth room‐2	Rbni^epi^‐At1	1	44.80	3.39	MM126	18.00	56.00	10.44	22.93	WT
		Growth room‐2	Rbni^epi^‐At4	4	42.43	3.72	MM691	34.00	50.00	11.51		WT
G‐ΔLf	Noninfected – Infected	Growth room‐2	ΔLf^epi^‐At1	1	20.00	3.79	MM10	16.45	52.00	10.44	31.55	*ddm1‐2*
		Growth room‐2	ΔLf^epi^‐At3	3	49.46	3.68	MM515	30.00	62.00	10.11		*ddm1‐2*
		Growth room‐2	ΔLf^epi^‐At5	5	47.36	3.08	MM854	37.80	54.00	8.38		*ddm1‐2*

Confidence intervals are in cM. Peak positions are indicated in cM and with the marker nearest to the logarithm of odds (LOD) score peak. R², phenotypic variation explained by the QTL^epi^. Chr, chromosome. For Lfi and Rbi, favourable alleles are associated with an increase in the value. For Pb, favourable alleles are associated with a decrease in the value. G‐Rbi, G‐Lfi and G‐Pb represent epigenetic QTL for each trait in infected condition. G‐Lfni and G‐Rbni represent epigenetic QTL for each trait in control condition. G‐ΔLf represents epigenetic QTL obtained by difference between leaf length in infected condition and leaf length in control condition. GT‐Rbi, GT‐Lfi and GT‐Pb represent epigenetic × temperature QTL for each trait.WT, wild‐type; *ddm1‐2*, mutant allele.DI, disease index; Pb, pathogen DNA quantity; Lfi, leaf length in infected condition; Rbi, root biomass in infected condition; Lfni and Rbni, respectively, leaf length and root biomass in control (noninfected) condition; ΔLf, change in leaf length.

#### QTL analysis of data from growth room‐2

As above, phenotypic data measured on the two biological replicates in growth room‐2 were used in glm1 to extract the epigenotype effect with the lsmean function. Once again, the epigenotype effect G identified for each trait was then used for the QTL^epi^ analysis. In total, seven QTL^epi^ were detected (Fig.**** 3). One QTL^epi^ was detected on chromosome 4 for Pb (Pb2^epi^‐At4) and two QTL^epi^ on chromosomes 1 and 3 were detected for Lfi (Lfi2^epi^‐At1, Lfi2^epi^‐At3). QTL mapping also revealed four QTL^epi^ controlling Rbi, which was measured only in this growth room: one on chromosome 1, two on chromosome 2 and one on chromosome 4 (Rbi^epi^‐At1, Rbi^epi^‐At2a, Rbi^epi^‐At2b, Rbi^epi^‐At4). Again, no QTL^epi^ was identified for DI despite a significant effect of the epigenotype. The variance explained by each fitted QTL model was of 10.86%, 26.16% and 44.59% for Pb, Lfi and Rbi, respectively. The QTL^epi^ identified for Pb on chromosome 4 explained 10.86% of the variability. The two QTL^epi^ detected on chromosomes 1 and 3 for Lfi explained (respectively) 15.65% and 12.06% of the phenotypic variation (Table 2). Confidence intervals of the detected QTL^epi^ ranged from 7.62 to 54.33 cM (Table**** 2). No epistatic interaction was found between QTL^epi^ for Lfi and Rbi traits. The additive allele effect of the two QTL^epi^ detected for Lfi (peak markers: MM91 and MM515) and of three of the four QTL^epi^ detected for Rbi (peak markers: MM147, MM383, MM686) was in the same direction: WT‐derived epialleles were associated with an increase in trait value. For the Pb QTL (peak marker: MM693) and one of the four QTL^epi^ of Rbi (peak marker: MM385), the *ddm1‐*derived epiallele was associated with a decrease in trait value (Fig. S1; Table 2).

For Lfi, QTL^epi^ detected on chromosomes 1 and 3 in growth room‐1 (Lfi1^epi^‐At1, Lfi1^epi^‐At3) co‐localized with the QTL^epi^ detected on chromosomes 1 and 3 in growth room‐2 (Lfi2^epi^‐At1, Lfi2^epi^‐At3). The QTL^epi^ on chromosome 5 was detected only in growth room‐1. The Pb QTL^epi^ detected in the two growth rooms were different. These results indicated that the QTL detection was possibly dependent on the growth room conditions (Fig. 3; Table 2).

As *ddm1* mutants displayed smaller roots and leaf lengths than WT in control conditions (i.e. noninoculated; Kakutani *et al*., 1996; Cortijo *et al*., 2014; Table S3), a similar experiment was carried out also in growth room‐2 without clubroot infection to determine the impact of developmental alteration due to the mutation on clubroot symptoms. The Lfni and Rbni data assessed on the 123 epiRIL in control condition showed continuous distribution, suggesting polygenic control of these traits (Fig. 2). Data analysis showed a significant epigenotype effect (glm1) on both phenotypic traits (Lfni: χ² = 15 771.8, *P‐*value < 2.2e‐16; Rbni: χ² = 33.09, *P‐*value = 3.48e‐10). In total, five QTL^epi^ were detected (Fig. 3): three QTL^epi^ were identified for Lfni on chromosomes 1, 3 and 5 (Lfni^epi^‐At1, Lfni^epi^‐At3 and Lfni^epi^‐At5) explaining 19.66%, 12.93% and 10.49% of the phenotypic variability, respectively. Two QTL^epi^ were detected for Rbni on chromosomes 1 and 4 (Rbni^epi^‐At1, Rbni^epi^‐At4) explaining 10.44% and 11.51% of phenotypic variation, respectively (Table 2). The additive allele effect of all the QTL^epi^ detected for Lfni (peak markers: MM126, MM515 and MM713) and Rbni (peak markers: MM126 and MM691) was in the same direction: WT‐derived epialleles were associated with an increase in trait value (Fig. S1). Three QTL^epi^ were detected with ΔLf on chromosomes 1, 3 and 5 (ΔLf^epi^‐At1, ΔLf^epi^‐At3 and ΔLf^epi^‐At5) explaining 10.44%, 10.11% and 8.38% of the phenotypic variability, respectively (Fig.**** 3). No QTL^epi^ was identified for ΔRb. The additive allele effect of all the QTL^epi^ detected for ΔLf (peak markers: MM10, MM515 and MM854) was also in the same direction but in this case the *ddm1‐2* epialleles were associated with the decrease of the value (Fig. S1).

### Temperature affects the plant response to *P. brassicae* in the epiRIL population

In order to explain the differences observed between the two growth rooms, we paid specific attention to the temperature conditions, as this parameter was shown to be critical for the development of clubroot disease (Siemens *et al*., 2002; Sharma *et al*., 2011). Although similar values of global mean temperature were noted in both growth rooms (growth room‐1 = 19.95°C, growth room‐2 = 20.06°C), significant differences (*F* = 2.67, *P‐*value = 0.002) in global temperature variances were registered between the two rooms (Fig. S2a). We then analysed mean and variance temperature values for each photoperiod (day and night). Day and night mean temperatures were similar in both chambers (growth room‐1: day temperature = 20.98°C, night temperature = 17.83°C; growth room‐2: day temperature = 20.85°C, night temperature = 18.51°C) and were very close to the required values (day temperature = 21°C, night temperature = 18°C). Conversely, the temperature variance in the two growth rooms and for both periods was significantly different (Fisher permutation test; day period: *F* = 9.05, *P‐*value = 0.002; night period: *F* = 3.67, *P‐*value = 0.002). The temperature range in growth room‐1 was 10.83°C for the day period and 4.98°C for the night period; the temperature range in growth room‐2 was 3.51°C for the day period and 1.88°C for the night period (Fig. S2b,c).

#### Effect of temperature variation on plant response to clubroot

Based on these observations, a more detailed analysis was carried out in growth room‐2 to evaluate the impact of the temperature variability on epiRIL response to clubroot. For this, we used data from temperature sensors placed at the height of the plants in growth room‐2. Significant differences (Kruskal–Wallis test) in the median temperatures were observed (χ² = 4115.3, df = 15, *P‐*value < 2.2e‐16) according to the location of the sensors. Pairwise analysis (Dunnett's test) of the differences between temperatures measured by the sensors showed significant temperature variations at several positions in the growth room (Table S5) and a temperature gradient from 21.4 to 24.1°C (Table S6).

A significant temperature effect (glm2) was shown for two of the phenotypic traits measured (*P‐*value ranged from 0.54 to < 2.2e‐16; Table S4). Moreover, a significant interaction between temperature and epigenotype effects (GT interaction) on the phenotypic traits measured was identified for nearly all traits (*P‐*value ranged from 0.44 to < 2.2e‐16; Table S4). Heritability (H²) in the case of temperature and epigenotype interaction was estimated for each trait using the formula (2.1). Heritability ranged from 0.50 to 0.67 according to the dataset used (Table 1).

#### Detection of epigenotype × temperature QTL^epi^

In order to determine whether temperature variation could influence plant response to clubroot in the epiRIL population, we associated each epiRIL with the mean temperature data measured with the temperature sensor placed in the block where the epiRIL was grown. These data were used in glm2 (Eqn (Eqn 2)) to extract the GT interaction using the lsmean function. The GT interaction values identified for each trait were then used for the QTL^epi^ analysis. Two QTL^epi^GT (epigenotype × temperature QTL^epi^) were detected on the chromosomes 1 and 3 for Lfi (Lfi^epi^GT‐At1, Lfi^epi^GT‐At3), and one QTL^epi^GT on chromosome 3 was detected for Rbi (Rbi^epi^GT‐At3). No QTL^epi^ was detected for Pb at a 5% significance level using the SIM by stepwise approach but two QTL^epi^ were detected on the chromosome 4 using a 10% significance level (Pb^epi^GT‐At4a, Pb^epi^GT‐At4b). Again, no QTL^epi^ could be detected for DI (Fig.**** 3). The variance explained by the fitted QTL model was 19.34% for Lfi, 9.06% for Rbi and 15.89% for Pb. The QTL^epi^ found for Lfi on chromosomes 1 and 3 explained 10.67% and 7.81% of the variability. The QTL^epi^ found for Rbi explained 9.06% of phenotypic variation. The two QTL^epi^ found for Pb on chromosome 4 explained 8.04% and 15.88% of the variation (Table 2). Confidence intervals of the detected QTL^epi^ ranged from 10.59 to 76 cM (Table**** 2). No epistatic interaction was found between QTL^epi^ for Lfi and Pb traits. For all QTL^epi^ detected, the WT epialleles were associated with an increase in the values (Fig. S1; Table 2). Comparison of the QTL^epi^ and QTL^epi^GT (taking into account the interaction with temperature) showed that all QTL^epi^GT co‐localized totally or partially with QTL^epi^ detected in at least one growth room (Fig. 3). Indeed, the comparison of the confidence intervals of QTL^epi^ detected using the leaf length trait showed that the QTL^epi^ Lfi1^epi^‐At1 and Lfi2^epi^‐At1 overlapped with the QTL^epi^GT Lfi^epi^GT‐At1. Likewise, Lfi1^epi^‐At3 and Lfi2^epi^‐At3 confidence intervals co‐localized with the confidence interval of Lfi^epi^GT‐At3 (Fig. 3; Table 2). For the QTL^epi^ detected using the quantification of the pathogen DNA (Pb) two overlaps were identified on chromosome 4, one between Pb1^epi^‐At4 and Pb^epi^GT‐At4a in the region extending from the short arm to the pericentromeric region and another between Pb2^epi^‐At4, Pb^epi^GT‐At4a and Pb^epi^GT‐At4b on the long arm of chromosome 4 (Fig. 3; Table 2).

### Impact of DNA sequence variation within QTL^epi^ confidence intervals

Although the epiRIL population was designed to minimize as much as possible DNA sequence variation, the presence of a small number of segregating nucleotidic variants cannot be avoided, notably as a result of the known effect of loss of DNA methylation on TE activity. In order to investigate the potential contribution of parental DNA sequence variants to the differential susceptibility to *P. brassicae* associated with the 20 QTL^epi^, we identified using whole genome sequencing data all sequence variants shared by more 25% of the epiRIL. In total, 63 small‐scale and 11 TE insertion variants were located within the 20 QTL^epi^, respectively. Eleven shared insertions of TE were detected in the QTL^epi^ confidence intervals, with 18 QTL^epi^ showing at least one insertion and two QTL^epi^ showing no insertion. All QTL^epi^ detected included at least one small‐scale sequence polymorphism in their confidence interval (Dataset S1).

Effects of the shared TEs and sequence variants were tested as described in Kooke *et al*. (2015). For 16 of 20 QTL^epi^, the effect of the epigenetic marker at the QTL peak was more significant than the effect of the TE or the small‐scale sequence variant (Dataset S2). However, for Lfi^epi^GT‐At1, Lfi^epi^GT‐At3, Lfi1^epi^‐At5 and ΔLf^epi^‐At3, the significant effect observed for a SNP or a TE was greater than for the epigenetic marker at the QTL peak (Dataset S2). In this case, we considered that these four QTL^epi^ were actually caused by DNA sequence variants. However, a linkage disequilibrium between the significant genetic markers and a causal epiallele could also be possible.

## Discussion

The aim of the present study was to investigate the role of epigenetic modifications in the Arabidopsis response to *Plasmodiophora brassicae*. To determine whether epigenetic regulation does play a role in clubroot quantitative resistance, a reverse genetics approach, using six T‐DNA insertional mutants (Col‐0 background) in genes involved in epigenetic pathways, was first carried out. For the six mutants tested, the disease index (DI) was only significantly reduced in the *ddm1* mutant compared to Col‐0. This finding suggests that *ddm1* confers decreased susceptibility of Arabidopsis to *P. brassicae*. This observation is in agreement with previous results obtained by Kellenberger *et al*. (2016) and Sharma *et al*. (2017) in the *Brassica* genus, which linked plant responses to biotic and abiotic stress to epigenetic regulations. Moreover, the involvement of *ddm1* in the Arabidopsis–*P. brassicae* interaction further supports the hypothesis that DNA methylation plays a role in plant pathogen infections (Dowen *et al*., 2012; López Sánchez *et al*., 2016; Hewezi *et al*., 2017). To decipher the epigenetic architecture of the clubroot resistance in Arabidopsis, we then carried out a epigenetic QTL (QTL^epi^) detection experiment using the epigenetic recombinant inbred lines (epiRIL) population. Four disease‐related traits (DI, pathogen DNA quantity (Pb), leaf length (Lfi) and root biomass (Rbi)), mostly used previously in Arabidopsis (Jubault *et al*., 2008; Gravot *et al*., 2011) to evaluate plant response to clubroot, were monitored. These traits were chosen in order to characterize disease development (disease index and pathogen DNA quantity) and the consequences of *P. brassicae* infection on plant development (Rbi and Lfi).

### Epigenetic QTL control Arabidopsis clubroot resistance

From our experiments using four biological replicates, we have shown a significant epigenetic effect on the response to *P. brassicae* infection. Thus, heritable plant responses induced by DNA methylation appear to be involved in the plant response to *P. brassicae*. The moderate to high heritability values (from 0.33 to 0.76) observed were similar to those described in control and abiotic stress conditions for this population (Johannes *et al*., 2009; Colomé‐Tatché *et al*., 2012; Kooke *et al*., 2015). Moreover, these heritability values also were similar to those described by Jubault *et al*. (2008) on a RIL population infected with *P. brassicae*. The moderate heritability levels calculated for the traits mean that this epigenetic variability could be considered for use in breeding. As the plant response to *P. brassicae* varied depending on the growth room used, we carried out the QTL^epi^ detection experiments using data from each growth room. In total, sixteen additive QTL^epi^ grouped in six genomic regions were identified distributed throughout four *A. thaliana* chromosomes. Among the 16 QTL^epi^, five QTL^epi^ were involved in pathogen multiplication (Pb) and 11 were involved in plant (foliar and root) development in response to *P. brassicae* infection. The identification of three QTL^epi^ for differences in longest length leaf (ΔLf) highlighted the modulation by *P. brassicae* infection of the foliar development variation already present in the epiRIL population (illustrated in Fig. S3). Clubroot resistance response is therefore composed of factors reducing the impact of the clubroot infection on foliar development as well as factors reducing pathogen development. For four of the 20 QTL^epi^ initially detected (Lfi^epi^GT‐At1, Lfi^epi^GT‐At3, Lfi^epi^‐At5 and ΔLf^epi^‐At3), analysis of shared transposition events (TE) and sequence variants included in their confidence intervals showed that the effect of DNA‐based markers was greater than the effect of epigenetic markers, suggesting that these QTL are not *bona fide* epigenetic. Among the four phenotypic traits evaluated, no QTL^epi^ was detected for DI despite a significant epigenotype effect in the two growth rooms (*P* = 1.57e‐06 and 1.65e‐04 for growth room‐1 and ‐2, respectively). Results of the reverse genetics approach showed that *ddm1* was less susceptible to *P. brassicae* compared to Col‐0 suggesting that *ddm1* epialleles confer a reduction in symptoms. In this context, the absence of QTL^epi^ detected for the DI trait may be explained by a sampling effect, because only a subset (123 of 505 lines) of the epiRIL population was phenotyped in this study, and/or a low proportion of *ddm1* epi‐haplotypes in the population subset (27%), which may have been insufficient to detect small QTL effects (Holland, 2007). Most of the QTL^epi^ detected in this study co‐localized with the pericentromeric regions. This may be explained by a more stable loss of methylation in those regions due to the loss of methylation maintenance on repeats and TE in *ddm1* (Kooke *et al*., 2015). Concerning the effect of the epialleles, the majority of the wild‐type (WT) epialleles were associated with an increase in the morphological trait values (length of leaves and root biomass). However, the *ddm1*‐derived epiallele led to a decrease in the amount of pathogen DNA for two of the three QTL^epi^ detected on chromosomes 1 and 4. This finding highlighted the positive effect of this epiallele on plant resistance, in agreement with the results obtained in the mutant test of this study and by Dowen *et al*. (2012) who showed a modest increase in *ddm1* resistance to *P. syringae*.

### Stability and pleiotropy of clubroot epigenetic QTL

Several QTL detected were stable across growth rooms. Indeed, despite the temperature variations between the two growth rooms, two overlapping leaf length QTL^epi^ (growth room‐1: Lfi1^epi^‐At1 and Lfi1^epi^‐At3; growth room‐2: Lfi2^epi^‐At1 and Lfi2^epi^‐At3) were detected in each growth room. Furthermore, for Pb, the QTL^epi^ Pb1^epi^‐At4, detected in the growth room‐1 overlapped with the QTL^epi^ Pb^epi^GT‐At4a detected in the growth room‐2 (Fig. 3; Table 2). The co‐localization on chromosome 1 of four QTL^epi^ controlling three different traits (Lfi, Pb and Rbi) and on chromosome 4 of three QTL^epi^ controlling two traits (Pb and Rbi) may suggest the presence of pleiotropic QTL^epi^ (Fig. 3; Table 2). However, analysis of the correlation (Spearman rho correlation) between traits showed only a moderate correlation (ρ = 0.52, *P* < 2.2e‐16) between Lfi and Rbi. Fine mapping is necessary to overcome the bias imposed by the large size of the QTL^epi^ confidence intervals and make further conclusions on the possibility of a pleiotropic gene or an effect due to linked genes.

### Temperature modulates Arabidopsis clubroot responses

Taking into consideration the temperature variations in growth room‐2, we tested the hypothesis that temperature plays a potential role in epiRIL and Col‐0 clubroot symptom variations. These observations are in agreement with the variations in clubroot severity observed on *Brassica rapa* subsp. *chinensis* and *B. napus* according to the temperature used for growing the plants (Sharma *et al*., 2011; Gossen *et al*., 2014). Siemens *et al*. (2002) had already evoked a possible link between temperature, clubroot response and epigenetics when studying the Arabidopsis mutant *tu8* (mutant in the *LIKE HETEROCHROMATIN PROTEIN 1 LHP1*) which presented different levels of response to clubroot depending on the temperature conditions. Similar environmental effects on the modulation of QTL controlling clubroot response also have been shown with nitrogen supply variations in *B. napus* (Laperche *et al*., 2017; Aigu *et al*., 2018) and with flooding in Arabidopsis (Gravot *et al*., 2016). Here, our analyses suggested that the temperature effect was partly triggered by the interaction with the plant epigenome for the traits Rbi and Pb. The identification of temperature‐dependent QTL^epi^ controlling pathogen quantity (Pb^epi^GT‐At4a and Pb^epi^GT‐At4b) suggests the presence of QTL^epi^ involved in the control of the pathogen development under temperature dependence. Similar observation concerning the detection of QTL temperature dependance was carried out by Aoun *et al*. (2017) using the model Arabidopsis–*Ralstonia solanacearum*. In their study, Aoun *et al*. (2017) showed that an increase of the temperature of 3°C during the interaction between Arabidopsis and *R. solanacearum* led to an increase of the sensitivity of most accessions and the loss of detection of one major QTL associated with the resistance. The influence of the temperature on the epiRIL response to *P. brassicae* could be explained by the increase of the environmental sensitivity triggered by the DNA hypomethylation suggested by Kooke *et al*. (2015).

### Clubroot resistance, a sophisticated system of regulation involving genetics and epigenetics

Interestingly, the comparison of the clubroot genetic QTL identified previously (Jubault *et al*., 2008) in Arabidopsis and the clubroot epigenetic QTL identified in this study highlighted overlapping of some confidence intervals. Indeed, the confidence intervals of six QTL^epi^ (Rbi^epi^‐At1, ΔLf^epi^‐ At1and Pb1^epi^‐At1, Lfi1^epi^‐At1, Lfi2^epi^‐At1, ΔLf^epi^‐At1) overlapped with two QTL (Pb‐At1 and Pb‐At4) found in the Bur‐0 × Col‐0 RIL population by linkage analysis (Jubault *et al*., 2008) and the major gene *RPB1* described by Arbeiter *et al*. (2002), respectively. These co‐localizations suggest that quantitative resistance to clubroot is modulated by a system involving both nucleotidic and epigenetic variations. These results illustrate the fact that in classical populations used for QTL detection, dissociation between causal genetic and epigenetic variations, both in linkage disequilibrium with markers, is nearly impossible (Schmitz *et al*., 2013a,b). In addition, our findings are consistent with the suggestion that during plant–pathogen interactions, plant transgenerational changes in genome structure and in DNA methylation patterns are possible (Boyko & Kovalchuk, 2011). The identification of two QTL^epi^ (Rbi^epi^‐At2 and Pb1^epi^‐At4) that did not show any overlap with previously reported QTL for these traits suggests that at these loci only epigenetic variation may be responsible for plant response variation. However, an absence of co‐localization could also be due to the absence of nucleotidic and/or epigenetic variations at these loci in the previously studied populations but does not exclude their existence in other genotypes.

This first study on the role of epialleles in the Arabidopsis–*P. brassicae* interaction brings to light the possibility of a complex model of quantitative resistance where alleles and epialleles act in concert. Furthermore, our study has shown that the temperature variations could influence epiRIL response to *P. brassicae*. In order to confirm whether epialleles are involved in plant response to clubroot infection, the QTL^epi^ confidence intervals must be reduced to find causal epialleles, notably through a fine‐mapping approach. The assessment of epiRIL in pathological tests in contrasting controlled temperature conditions is also needed to validate the possible role of temperature in modulating the epigenetic plant response to clubroot infection.

## Author contributions

BL, EJ, AE, MJ, AG, JL and CL carried out the experiments and collected the data; BL, JL and CL carried out the molecular biology work; BL and VB carried out the genetic analyses; BL and AE carried out the bioinformatics analyses; VB, ME and VC provided DNA sequence information about the epiRIL used in this study; BL, VB, MJM‐D and MJ wrote the article, assisted by AG and VC for manuscript editing; and BL, MJM‐D and MJ designed and coordinated the study.

## Supporting information

Please note: Wiley Blackwell are not responsible for the content or functionality of any Supporting Information supplied by the authors. Any queries (other than missing material) should be directed to the *New Phytologist* Central Office.

**Dataset S1** Location of segregating DNA sequence variants (SNP, indel, TE, SV) among the clubroot QTL intervals.

**Dataset S2 **Statistic analysis of TE and sequence variant effect compared to methylated markers on QTL^epi^ detection for each trait study.

**Fig. S1 **Epiallele effects at the closest QTL^epi^ peak marker.
**Fig. S2 **Boxplot of temperature data recorded in each growth room.
**Fig. S3 **Comparison of the epiallele effects at the closest QTL^epi^ peak marker between Lfi and Lfni in growth chamber‐2.
**Table S1 **List of primer sets used to confirm homozygosity of the T‐DNA insertion in mutants.
**Table S2** Disease index for Col‐0 and mutants after infection by *P. brassicae*, and Dunnett's post hoc test results.
**Table S3 **Phenotypic responses of epiRIL and their parent lines to infection by *P. brassicae*.
**Table S4** Analysis of epigenotype, temperature and interaction between temperature and epigenotype effect for each trait in infected condition.
**Table S5** Dunnett's test comparison of temperatures recorded by each temperature sensor in growth room‐2.
**Table S6** Median temperature monitored by each temperature sensor in growth room‐2.
