# Supplementary material for: Quantitative resistance to clubroot infection mediated by transgenerational epigenetic variation in Arabidopsis
Source: New Phytol. 2018 Nov 30;222(1):468–79. doi: 10.1111/nph.15579 (PMC6587750; doi:10.1111/nph.15579)
Supplement: Supplementary file 3 — Fig. S1 Epiallele effects at the closest QTLepi peak marker. Fig. S2 Boxplot of temperature data recorded in each growth room. Fig. S3 Comparison of the epiallele effects at the closest QTLepi peak marker between Lfi and Lfni in growth chamber‐2. Table S1 List of primer sets used to confirm homozygosity of the T‐DNA insertion in mutants. Table S2 Disease index for Col‐0 and mutants after infection by P. brassicae, and Dunnett's post hoc test results. Table S3 Phenotypic responses of epiRIL and their parent lines to infection by P. brassicae. Table S4 Analysis of epigenotype, temperature and interaction between temperature and epigenotype effect for each trait in infected condition. Table S5 Dunnett's test comparison of temperatures recorded by each temperature sensor in growth room‐2. Table S6 Median temperature monitored by each temperature sensor in growth room‐2. [file NPH-222-468-s003.pdf]

New Phytologist **Supporting Information**

Article title: Quantitative resistance to clubroot infection mediated by transgenerational epigenetic variation in *Arabidopsis*

Authors: Benjamin Liégard, Victoire Baillet, Mathilde Etcheverry, Evens Joseph, Christine Lariagon, Jocelyne Lemoine, Aurélie Evrard, Vincent Colot, Antoine Gravot, Maria J. Manzanares-Dauleux, Mélanie Jubault.

Article acceptance date: 26 October 2018

The following Supporting Information is available for this article:

Fig. S1. Epi-allele effects at the closest QTL^{epi} peak marker.

Fig. S2. Boxplot of temperature data recorded in each growth room.

Fig. S3. Comparison of the epi-allele effects at the closest QTL^{epi} peak marker between Lfi and Lfni in growth chamber 2.

Table S1: List of primer sets used to confirm homozygosity of the T-DNA insertion in mutants.

Table S2. (a) Disease index for Col-0 and mutants after infection by *Plasmodiophora brassicae*. (b) Dunnett post hoc test results.

Table S3. Phenotypic responses of epiRIL and their parent lines to infection by *Plasmodiophora brassicae*.

Table S4. Analysis of epigenotype, temperature and interaction between temperature and epigenotype effect for each trait in infected condition.

Table S5: Dunnett test comparison of temperatures recorded by each temperature sensor in growth room-2.

Table S6. Median temperature monitored by each temperature sensor in growth room-2.

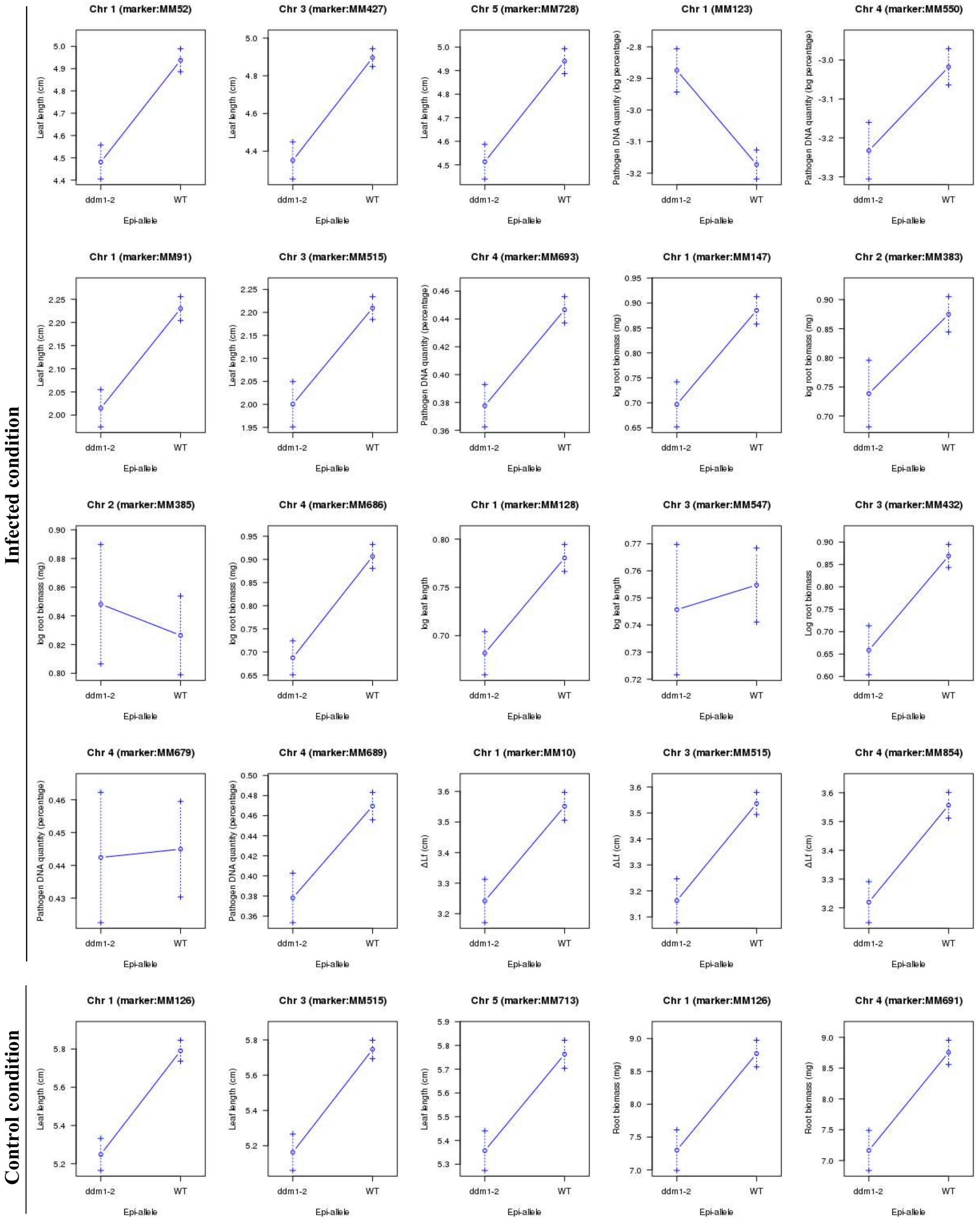

Fig. S1. Epi-allele effects at the closest QTL^{epi} peak marker. All graphics show the effect of each epi-allele on the trait value at the closest QTL peak marker. For all QTL^{epi} detected for root biomass, leaf length, pathogen quantity and ΔLf in growth room-2, the *ddm1-2* epi-allele is associated with a lower value than the WT epi-allele. For all QTL detected for the pathogen quantity in growth room-1, the *ddm1-2* epi-allele is associated with a higher value than the WT epi-allele.

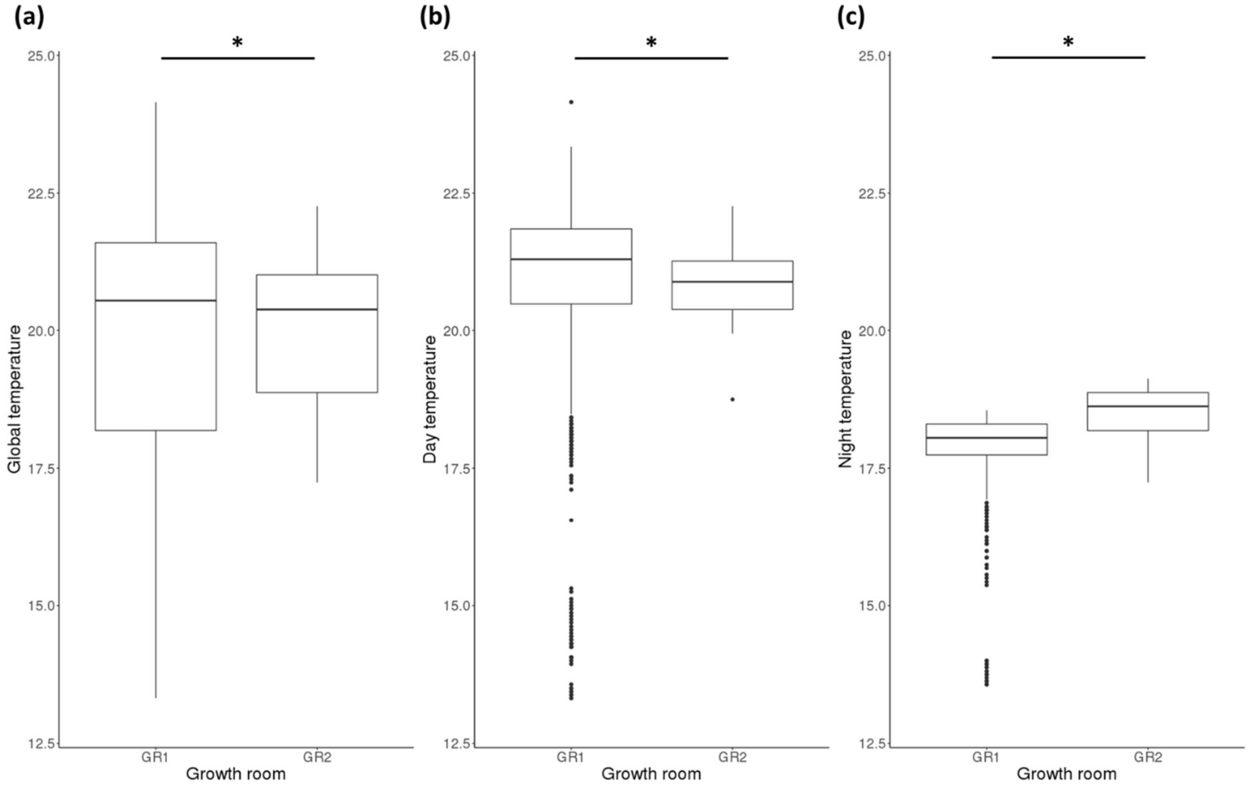

Fig. S2. Boxplot of temperature data recorded in each growth room. (a) Global temperature in each growth room. Stars indicate the significant difference (Fisher permutation test, F = 2.67, *p-value* = 0.002) of the variance of the global temperature between the two growth rooms. (b) Day temperature in each growth room. Stars indicate the significant difference (Fisher permutation test, F = 9.05, *p-value* = 0.002) of the variance of the day temperature between the two growth rooms. (c) Night temperature in each growth room. Stars indicate the significant difference (Fisher permutation test, F = F = 3.67, *p-value* = 0.002) of the variance of the day temperature between the two growth rooms. Boxplot showed the median (inner box black horizontal line), the 25 and the 75 percentiles (box), the 1.5 times interquartile (vertical line) and the extreme values (black dots).

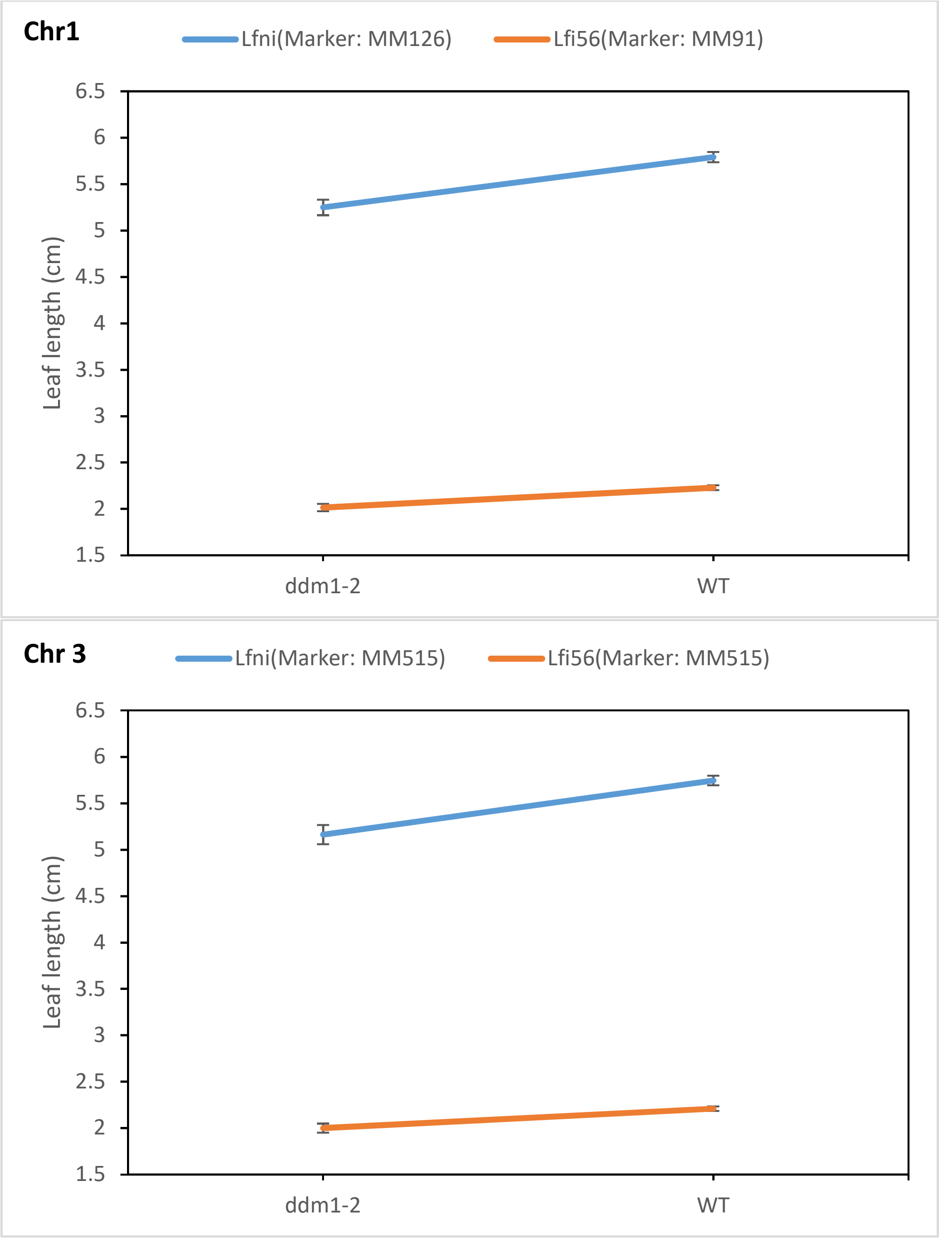

Fig. S3. Comparison of the epi-allele effects at the closest QTL^{epi} peak marker between Lfi and Lfni in growth chamber 2. The effect of each epi-allele at the closest QTL peak marker on the trait value is shown for the leaf length in infected condition and control condition. For the two QTL^{epi} detected on the chromosomes 1 and 3 in inoculated and inoculated conditions, the difference between the effect of the epiallele WT and the epiallele *ddm1-2* is less important in infected condition than in control condition, illustrating the modulation of the epigenetic effect on the leaf length by the infection.

Table S1. List of primer sets used to confirm homozygosity of the T-DNA insertion in mutants.

NASC number	Mutant	Gene	LP primer (5' > 3')	RP primer (5' > 3')
N650863	SALK_150863	At5g14620	AGATCGCTTCCAGAGTTAGCC	TTGTCGCAAAAAGCAAAAGAG
N655230	SALK_004027C	At3g18520	CTTCTCTGTTCATCGTTTCGC	AGCAACATTCTCTCGTCGAAC
N667337	SALK_130607C	At5g09790	TTTCTCTTGTCCGGTGAAATG	CCTGCAACAATCAGTGTGATG
N681376	SALK_082118C	At1g79000	GACTGACAGGTCTTTGCTTGG	ATGAACCTGGAATGGAGAACC
N681865	SALK_149295C	At5g09230	CGCAGAGAGAGAACAAAATCG	TTCCACATTCTGTGCTAACCC
N681885	SALK_000590C	At5g66750	GAAGACAGGTTTCACCTGTGC	CGTGAGAAATAGCTCAATGCC

Table S2. (a) Disease index for Col-0 and mutants after infection by *Plasmodiophora brassicae*. (b) Dunnett post hoc test results.

(a)

Lines	Means	Sd
ddm1 *	46.56	14.63
srt2	61.67	12.58
drm2	66.67	14.43
atxr5	70.28	15.28
hac1	70.56	18.96
**Col-0**	77.09	8.23
hdc15	84.72	19.7

Disease index calculation was carried out according to Manzanares-Dauleux *et al.* (2000b). Means were obtained by averaging the data from three blocks with six plants by block. Standard deviation (Sd) was obtained from the difference in DI between the three blocks for each mutant and Col-0. The star next to *ddm1* indicates significant differences for the disease index compared to Col-0.

(b)

DI genotype comparison	Estimate	Std.Error	t value	Pr(>|t|)
atxr5 - Col-0	-6.8083	6.880	-0.990	0.86361
ddm1 - Col-0	-30.5283	6.880	-4.437	0.00269
drm2 - Col-0	-10.4183	6.880	-1.514	0.53771
hac1 - Col-0	-6.5283	6.880	-0.949	0.88306
hdc15 - Col-0	7.6383	6.880	1.110	0.79812
srt2 - Col-0	-15.4183	6.880	-2.241	0.18262

Test was carried using multcomp R package.

Table S3. Phenotypic responses of epiRIL and their parent lines to infection by *Plasmodiophora brassicae*.

Lines	Condition	Growth room	Leaf length (cm)	Root biomass (mg per plant)	Pathogen DNA quantity (%)	Disease index
Col-0	Infected	1	5.52 ± 0.59	NA	0.036 ± 0.01	53.25 ± 2.36
		2	1.98 ± 0.34	1.95 ± 0.23	0.45 ± 0.05	90.75 ± 9.64
	Control	2	5.98 ± 0.39	14.12 ± 4.43	NA	NA
ddm1-2	Infected	1	NA	NA	NA	NA
		2	2.12 ± 0.54	2.16 ± 0.72	0.31 ± 0.13	71.75 ± 17.11
	Control	2	4.28 ± 0.10	8.17 ± 2.84	NA	NA
Epirils	Infected	1	4.80 ± 0.75	NA	0.052 ± 0.04	51.17 ± 11.42
		2	2.17 ± 0.47	2.41 ± 0.95	0.43 ± 0.17	86.27 ± 10.74
	Control	2	5.63 ± 0.78	8.36 ± 3.64	NA	NA

Disease index calculation was carried out according to Manzanares-Dauleux *et al.* (2000). Pathogen DNA quantification was determined by quantitative PCR. For each trait, condition and growth room, means were obtained by averaging data of two biological replicates, each one designed with two blocks and six plants per block. Standard deviation (Sd) was obtained from the difference in each trait between the four blocks. NA stands for non available.

Table S4. Analysis of epigenotype, temperature and interaction between temperature and epigenotype effect for each trait in infected condition. Effect was estimated using the (glm, model 1) for the G effect and using the glm (model 2) for the temperature effect and GT effect.

Trait measured	Growth room	Epigenotype effect (G)	Interaction between temperature and epigenotype effect (GT)	Temperature effect
Lfi	GR1	$\chi^2 = 126.32$, *p-value* <2.2e- 16	NA	NA
	GR2	$\chi^2 = 32.74$, *p-value* <2.2e-16	$\chi^2 = 23.58$, p-value <2.2e-16	$\chi^2 = 10.14$, *p-value* <2.2e-16
DI	GR1	$\chi^2 = 22256.3$, *p-value* = 1.57e-06	NA	NA
	GR2	$\chi^2 = 2.69$, *p-value* = 1.65e-4	DI: $\chi^2 = 1.79$, *p-value*= 0.44	$\chi^2 = 0.005$, *p-value*= 0.54
Pb	GR1	$\chi^2 = 71.73$, *p-value* = 0.02	NA	NA
	GR2	$\chi^2 = 3.75$, p-value = 0.35	Pb: $\chi^2 = 4.20$, p-value = 0.01	$\chi^2 = 0.01$, *p-value* = 0.53
Rbi	GR1	NA	NA	NA
	GR2	$\chi^2 = 28.05$, *p-value* <2.2e-16	Rbi: $\chi^2 = 14.51$, p-value = 0.002	$\chi^2 = 2.98$, *p-value* = 2.94e-09

Table S5. Dunnett test comparison of temperatures recorded by each temperature sensor in growth room-2.

	**Ts1**	**Ts2**	**Ts3**	**Ts4**	**Ts5**	**Ts6**	**Ts7**	**Ts8**	**Ts9**	**Ts10**	**Ts11**	**Ts12**	**Ts13**	**Ts14**	**Ts15**
**Ts2**	< 2.00E-16	_	_	_	_	_	_	_	_	_	_	_	_	_	_
**Ts3**	< 2.00E-16	6.50E-06	_	_	_	_	_	_	_	_	_	_	_	_	_
**Ts4**	< 2.00E-16	1.10E-04	< 2.00E-16	_	_	_	_	_	_	_	_	_	_	_	_
**Ts5**	< 2.00E-16	< 2.00E-16	< 2.00E-16	< 2.00E-16	_	_	_	_	_	_	_	_	_	_	_
**Ts6**	< 2.00E-16	< 2.00E-16	< 2.00E-16	< 2.00E-16	**1.00**	_	_	_	_	_	_	_	_	_	_
**Ts7**	< 2.00E-16	< 2.00E-16	< 2.00E-16	< 2.00E-16	**5.90E-02**	2.90E-06	_	_	_	_	_	_	_	_	_
**Ts8**	< 2.00E-16	< 2.00E-16	< 2.00E-16	< 2.00E-16	1.77E-0.2	**1.00**	2.90E-06	_	_	_	_	_	_	_	_
**Ts9**	2.6 E-16	< 2.50E-06	**1.00**	< 2.00E-16	<2.00E-16	< 2.00E-16	< 2.00E-16	< 2.00E-16	_	_	_	_	_	_	_
**Ts10**	< 2.00E-16	< 2.00E-16	< 2.00E-16	3.50E-05	6.40E-13	< 2.00E-16	2.30E-04	< 2.00E-16	<2.00E-16	_	_	_	_	_	_
**Ts11**	< 2.00E-16	**1.00**	3.60E-08	2.54E-02	<2.00E-16	< 2.00E-16	< 2.00E-16	< 2.00E-16	1.50E-08	1.70E-14	_	_	_	_	_
**Ts12**	< 2.00E-16	< 2.00E-16	< 2.00E-16	3.90E-12	2.50E-06	2.20E-12	**1.00**	<2.00E-16	< 2.00E-16	**1.00**	< 2.00E-16	_	_	_	_
**Ts13**	< 2.00E-16	< 2.00E-16	< 2.00E-16	< 2.00E-16	2.5E-16	1.70E-09	<2.00E-16	7.40E-06	< 2.00E-16	< 2.00E-16	< 2.00E-16	< 2.00E-16	_	_	_
**Ts14**	< 2.00E-16	< 2.00E-16	< 2.00E-16	< 2.00E-16	4.80E-12	3.10E-06	<2.00E-16	2.68E-03	< 2.00E-16	< 2.00E-16	< 2.00E-16	< 2.00E-16	**1.00**	_	_
**Ts15**	< 2.00E-16	< 2.00E-16	< 2.00E-16	2.50E-14	5.80E-06	3.90E-12	**1.00**	< 2.00E-16	< 2.00E-16	**4.29E-01**	< 2.00E-16	**1.00**	< 2.00E-16	< 2.00E-16	_
**Ts16**	< 2.00E-16	< 2.00E-16	< 2.00E-16	8.60E-13	4.60E-07	1.20E-13	**7.89E-01**	< 2.00E-16	< 2.00E-16	**1.00**	< 2.00E-16	**1.00**	< 2.00E-16	< 2.00E-16	**1.00**

Adjusted *p-values* (Bonferroni method) calculated with the Dunnett test between each temperature sensor are presented in the bottom left of the table. Ts corresponds to the temperature sensor number. Non-significant *p-values* of test were highlighted in bold.

Table S6. Median temperature monitored by each temperature sensor in growth room-2.

Temperature sensor	Median temperature	MAD
Ts1	21.40	0.74
Ts2	22.90	0.74
Ts3	22.50	0.74
Ts4	22.90	0.74
Ts5	23.20	0.74
Ts6	23.60	0.74
Ts7	23.10	0.74
Ts8	23.20	1.11
Ts9	22.10	0.74
Ts10	23.10	0.74
Ts11	22.60	0.74
Ts12	23.20	0.74
Ts13	24.10	0.74
Ts14	23.70	0.74
Ts15	23.10	0.74
Ts16	23.10	0.74

MAD: Median absolute deviation. The median temperatures are in °C. Ts correspond to the temperature sensor number.

REFERENCES

Manzanares-Dauleux MJ, Divaret I, Baron F, Thomas G. 2000. Evaluation of French *Brassica oleracea* landraces for resistance to *Plasmodiophora brassicae*. *Euphytica* **113**(3): 211-218.
